# Comparative and Phylogenetic Analysis of Complete Chloroplast Genomes in Eragrostideae (Chloridoideae, Poaceae)

**DOI:** 10.3390/plants10010109

**Published:** 2021-01-06

**Authors:** Kuan Liu, Rong Wang, Xiu-Xiu Guo, Xue-Jie Zhang, Xiao-Jian Qu, Shou-Jin Fan

**Affiliations:** Shandong Provincial Key Laboratory of Plant Stress Research, College of Life Science, Shandong Normal University, No. 88 Wenhuadong Road, Jinan 250014, China; 2018020778@stu.sdnu.edu.cn (K.L.); 2017010077@stu.sdnu.edu.cn (R.W.); 2018010081@stu.sdnu.edu.cn (X.-X.G.); 109131@sdnu.edu.cn (X.-J.Z.)

**Keywords:** *Eragrostis*, Eragrostideae, chloroplast genome, comparative genomics, phylogenomics

## Abstract

Eragrostideae Stapf, the second-largest tribe in Chloridoideae (Poaceae), is a taxonomically complex tribe. In this study, chloroplast genomes of 13 Eragrostideae species were newly sequenced and used to resolve the phylogenetic relationships within Eragrostideae. Including seven reported chloroplast genomes from Eragrostideae, the genome structure, number and type of genes, codon usage, and repeat sequences of 20 Eragrostideae species were analyzed. The length of these chloroplast genomes varied from 130,773 bp to 135,322 bp. These chloroplast genomes showed a typical quadripartite structure, including a large single-copy region (77,993–80,643 bp), a small single-copy region (12,410–12,668 bp), and a pair of inverted repeats region (19,394–21,074 bp). There were, in total, 129–133 genes annotated in the genome, including 83–87 protein-coding genes, eight rRNA genes, and 38 tRNA genes. Forward and palindromic repeats were the most common repeat types. In total, 10 hypervariable regions (*rpl22*, *rpoA*, *ndhF*, *matK*, *trnG*–*UCC-trnT*–*GGU*, *ndhF*–*rpl32*, *ycf4*–*cemA*, *rpl32*–*trnL*–*UAG*, *trnG*–*GCC*–*trnfM*–*CAU*, and *ccsA*–*ndhD*) were found, which can be used as candidate molecular markers for Eragrostideae. Phylogenomic studies concluded that *Enneapogon* diverged first, and *Eragrostis* including *Harpachne* is the sister to *Uniola*. Furthermore, *Harpachne harpachnoides* is considered as a species of *Eragrostis* based on morphological and molecular evidence. In addition, the interspecies relationships within *Eragrostis* are resolved based on complete chloroplast genomes. This study provides useful chloroplast genomic information for further phylogenetic analysis of Eragrostideae.

## 1. Introduction

Chloroplasts are the organelles necessary for photosynthesis, and the most important and common plastids in plant cells. In addition, chloroplasts are semi-autonomous organelles with their own genome, which is a relatively independent genetic system in plant cells. Compared with the nuclear genome, the chloroplast genome has the characteristics of small size, single parent inheritance, low nucleotide substitution rate, and highly conserved genome structure. In angiosperms, the chloroplast genome is relatively conservative, and has a typical quadripartite structure with a pair of inverted repeat regions (IR_b_/IR_a_), a large single copy (LSC) region, and a small single copy (SSC) region [1]. The length of the chloroplast genome varies greatly from species to species [2]. With the rapid development of next-generation sequencing (NGS) technology, it is easier to obtain the complete chloroplast genome, making chloroplast genomes a research hotspot [3,4,5]. The chloroplast genome has important value in studying the phylogeny of species [6,7]. The research scope of chloroplast genomes is also relatively wide, including comparative genomics research, phylogenetic research, and simple sequence repeat (SSR) genetic polymorphism research [8,9].

Due to the limited taxonomic traits that can be reflected by herbarium specimens, Chloridoideae has always been one of the most difficult groups to study in Poaceae systematics [10,11]. Peterson et al., (2010) [12] divided Chloridoideae into four tribes (Triraphideae, Eragrostideae, Zoysieae, and Cynodonteae) based on multiple gene sequences. In Eragrostideae, Cotteinae (including *Cottea* and *Enneapogon*) diverged first, and Eragrostidinae (including *Ectrosia*, *Harpachne*, *Psammagrostis*, and *Eragrostis*) is the sister to Uniolinae (including *Entoplocamia*, *Tetrachne*, and *Uniola*). Eragrostideae Stapf is the second-largest and more complex tribe in Chloridoideae. There are about 500 species in this tribe [13,14]. All species in Eragrostideae use the C_4_ photosynthetic pathway (*Eragrostis walteri* is a C_3_ plant [15,16]), and most of them are distributed in tropical and subtropical regions [14,17]. Members of Eragrostideae are generally characterized by laterally compressed spikelets, glabrous three (to 13)-nerved lemmas, and ciliate ligule [18].

*Eragrostis* Wolf, the type genus of Eragrostideae, is the largest genus in Eragrostideae. There are more than 400 species worldwide [19,20]. Due to its large number of species, diverse chromosome ploidy, and similar morphological characteristics between species [21], it is a complex genus in Eragrostideae. Due to its large size and wide geographical distribution, comprehensive taxonomic treatment of the genus remains difficult. Several phylogenetic studies focusing on *Eragrostis* and its related genera have been carried out [3,21,22,23], however, correct intergeneric and infrageneric relationships still remain unresolved. There has been some debate in the recent literature as to whether the genus is monophyletic. Several studies [3,21] suggested that *Eragrostis* was a monophyletic group, however, *Eragrostis* is considered to be a paraphyletic group in some studies [24,25]. Ingram and Doyle [23] found that *Eragrostis* was a monophyletic group with the inclusion of four segregate genera: *Acamptoclados*, *Diandrochloa*, *Neeragrostis*, and *Pogonarthria* based on the plastid locus *rps16* and nuclear gene *waxy*. Peterson et al. [12] found a terminal Eragrostidinae clade of *Ectrosia*, *Harpachne*, and *Psammagrostis* embedded in a polyphyletic *Eragrostis*. In addition, the infrageneric relationships of *Eragrostis* could not be solved based on partial molecular sequences [21,22]. However, Somaratne et al. [3] reconstructed the relationships among the five species of *Eragrostis*, according to the whole chloroplast genomes. Therefore, the above shows that more evidence and broader sampling are needed to resolve the phylogenetic relationships within *Eragrostis*. *Harpachne* Hochst. was first recorded in 1841, and *Harpachne harpachnoides* (Hack.) B. S. Sun and S. Wang was described as “*Eragrostis harpachnoides* Hack.” in Oesterreichische Botanische Zeitschrift. in 1902, which shows that *Harpachne* is closely related to *Eragrostis*. *Harpachne* is a small genus that contains only three species: *Harpachne bogdanii* Kenn.-O’Byrne, *Harpachne harpachnoides* (Hack.) B. S. Sun and S. Wang, and *Harpachne schimperi* A. Rich. In Flora of China [17], *Harpachne* is distinguished from *Eragrostis* by the morphology of inflorescence, but both have ciliated ligules and three-veined lemmas. Peterson et al. [12] found that *Harpachne* was embedded in *Eragrostis* based on multi-gene phylogenetic trees, which was consistent with previous studies [13,24,25] based on a few molecular sequences. Reconstruction of the phylogenetic relationship between *Eragrostis* and *Harpachne* through complete chloroplast genomes has not been previously reported. Therefore, whether *H. harpachnoides* was a species of *Eragrostis* can be studied with both morphological and complete chloroplast genome evidence.

**Figure 1:**
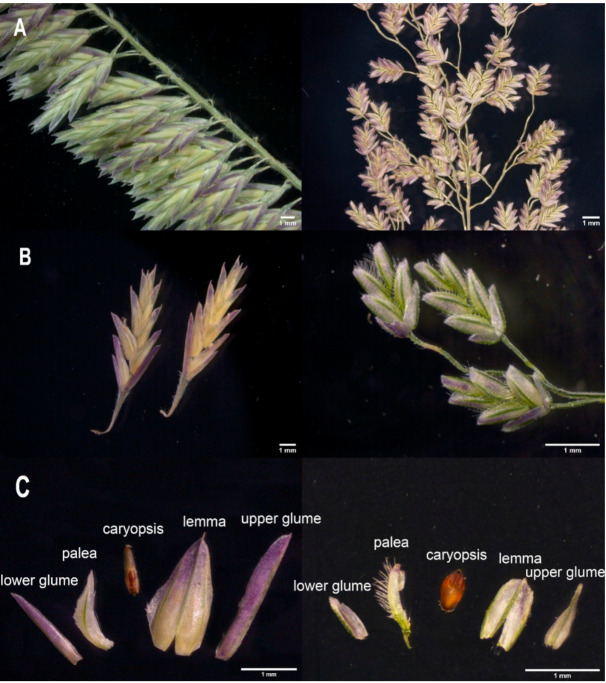
The flower structures of *Harpachne harpachnoides* (left) and *Eragrostis tenella* (right). (**A**) Inflorescence structure; (**B**) Spikelets structure; (**C**) Floret structure.

In this study, chloroplast genomes of 11 *Eragrostis* species, *Enneapogon desvauxii*, and *Harpachne harpachnoides* were newly sequenced (Table 1). A genomic comparative analysis was performed in combination with chloroplast genomes of four other *Eragrostis* species, one *Uniola* species, and two other *Enneapogon* species available in GenBank. In addition, we carried out anatomy investigations of the spikelets of *H. harpachnoides* and *E. tenella*, and compared their morphological difference (Figure 1). The main purpose of this study was to: (1) compare and analyze the chloroplast genome structure of the 20 Eragrostideae species; (2) identify highly divergent regions of all 20 Eragrostideae chloroplast genomes; (3) explore the phylogenetic position of *Harpachne* relative to *Eragrostis*, and resolve the interspecies relationships within *Eragrostis*. In summary, this study will provide important insight in understanding the chloroplast genome evolution and phylogeny of Eragrostideae species.

## 2. Results

### 2.1. Chloroplast Genome Characteristics of Eragrostideae

The complete chloroplast genome length of the 20 Eragrostideae species varied from 130,773 bp (*Eragrostis tenellula*) to 135,322 bp (*Uniola paniculata*), and showed a typical quadripartite structure with the LSC region (77,993–80,643 bp), SSC region (12,410–12,668 bp), and a pair of IR regions (19,394–21,074 bp) (Table 2). The overall guanine–cytosine (GC) content of each species was approximately 38% (Table 2). The GC content in the IR region was higher than both the LSC and SSC regions. There were 129–133 genes, including 83–87 protein-coding genes (PCGs), eight ribosomal RNA genes (rRNAs), and 38 transfer RNA genes (tRNAs) (Table 2). *E. tenellula* had the fewest genes, and lacked *rps15*. We found that there were some conserved *ycf1* and *ycf2* gene residues in some species of Eragrostideae, and the *accD* gene had completely degraded. In addition, the intron sequences of the *clpP* gene and the *rpoC1* gene had been lost. Therefore, there were 16 intron-containing genes in each of the Eragrostideae species, of which, two PCGs (*ycf3* and *rps12*) had two introns, and eight PCGs (*ndhB*, *rpl2*, *ndhA*, *rpl16*, *petB*, *atpF*, *petD*, and *rps16*) and six tRNAs (*trnA-UGC*, *trnI-GAU*, *trnK-UUU*, *trnG-UCC*, *trnV-UAC*, and *trnL-UAA*) had a single intron.

### 2.2. Repeat Sequences and SSRs Analysis

**Figure 2:**
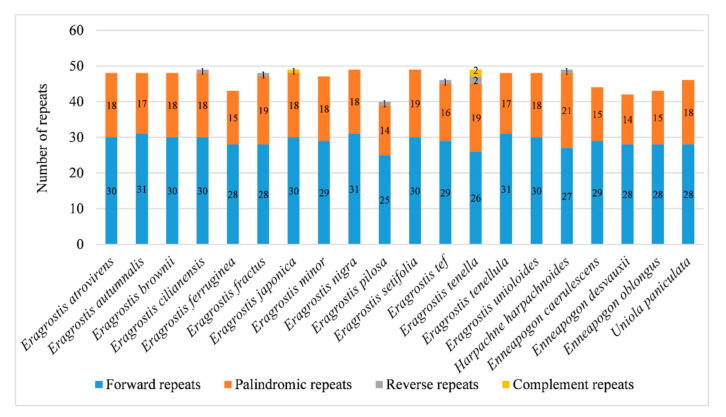
The number of four repeat types in 20 Eragrostideae chloroplast genomes.

**Figure 3:**
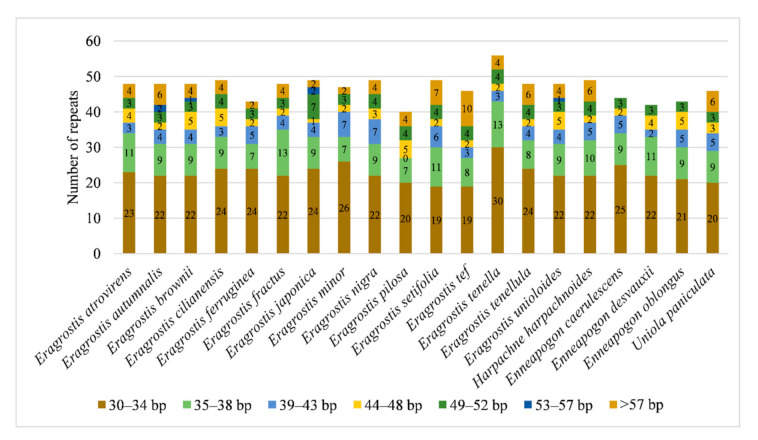
The number of repeats with different length in 20 Eragrostideae chloroplast genomes.

A total of 933 repeats were identified in 20 Eragrostideae chloroplast genomes through REPuter, including 578 forward repeats, 345 palindromic repeats, seven reverse repeats, and three complementary repeats. Each species detected 47 repeats on average. *E. cilianensis*, *E. japonica*, *E. nigra*, *E. setifolia*, *E. tenella*, and *H. harpachnoides* had the largest number of repeats (49), while *E. pilosa* had the smallest number of repeats (40; Figure 2). Three complementary repeats were only detected in the chloroplast genomes of *E. japonica* (one) and *E. tenella* (two). The length of the repeats was mainly concentrated in 30–38 bp (Figure 3).

A total of 943 SSRs were identified in the chloroplast genomes of 20 Eragrostideae species using MISA script. These SSRs were mainly distributed in the LSC region (Table 3). These SSRs were mononucleotide repeats and dinucleotide repeats, and the mononucleotide repeat type was mainly A/T repeat, while all the dinucleotide repeat sequences were composed of AT/TA repeats (Table 3). There were no trinucleotide or longer repeats in these chloroplast genomes. *E. tenella* had the largest number of SSRs (56), including 53 mononucleotide repeats (51 A/T repeats and two G/C repeats) and three dinucleotide repeats. *E. atrovirens* and *E. setifolia* both had the minimum number of SRRs (40). The SSRs in *U. paniculata* were all distributed in the LSC region. SSRs were not found in the SSC region in *E. desvauxii*. In these species, compound SSRs were also rich in A/T repeats, and all were located in the LSC region.

### 2.3. Codon Usage Analysis

**Figure 4:**
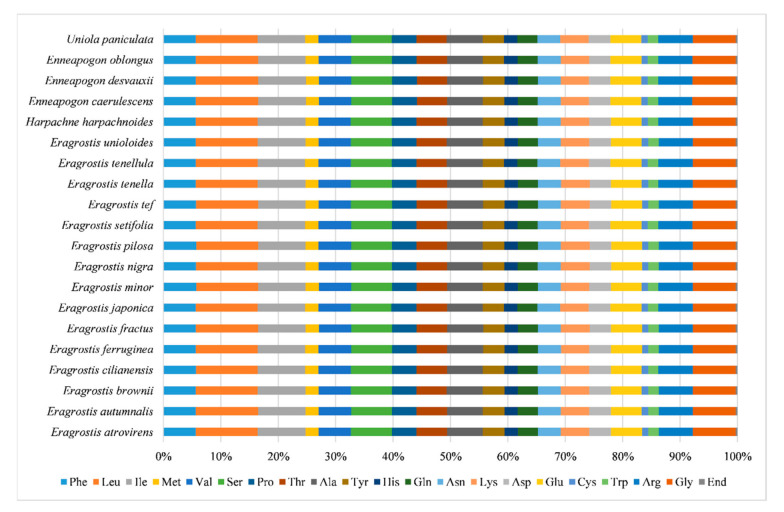
Amino acid proportion of protein-coding genes in 20 Eragrostideae chloroplast genomes.

By removing repeats and lengths of less than 300 bp sequences, 51 coding sequences (CDSs) were selected from 16 Eragrostideae species, and 50 CDSs were selected from four Eragrostideae species (*E. japonica*, *E. ferruginea*, *E. tenellula*, and *U. paniculata*). The analysis results showed that the variation range of the total GC content was 38.9–39.1%, with an average value of 38.97%. The result showed that the GC content was low and the difference in content between various species was not significant. The number of codons ranged from 16,999 (*E. japonica*) to 17,210 (*E. pilosa*), with an average value of 17,151 (Table 4). Among them, there were six codon types encoding leucine, serine, and arginine, while there was only one codon type encoding methionine and tryptophan (Table S1). In addition, leucine was the most amino acid encoded in the chloroplast genome, accounting for 10.82% on average of all amino acids. Cysteine had the smallest number of codons, accounting for only 1.09% on average of all amino acids (Figure 4). The values for the effective number of codons (ENC) in Eragrostideae chloroplast genomes ranged from 49.40 to 49.80, with an average value of 49.56 (Table 4). In all species, there were 31 codons with an relative synonymous codon usage (RSCU) value greater than 1.00, of which, 29 ended with A or U codons and two ended with G or C codons (UUG, UGU). In addition, the RSCU value of methionine (AUG) and tryptophan (UGG) was 1.00 (Table S2).

### 2.4. Expansion and Contraction of the IR Region

**Figure 5:**
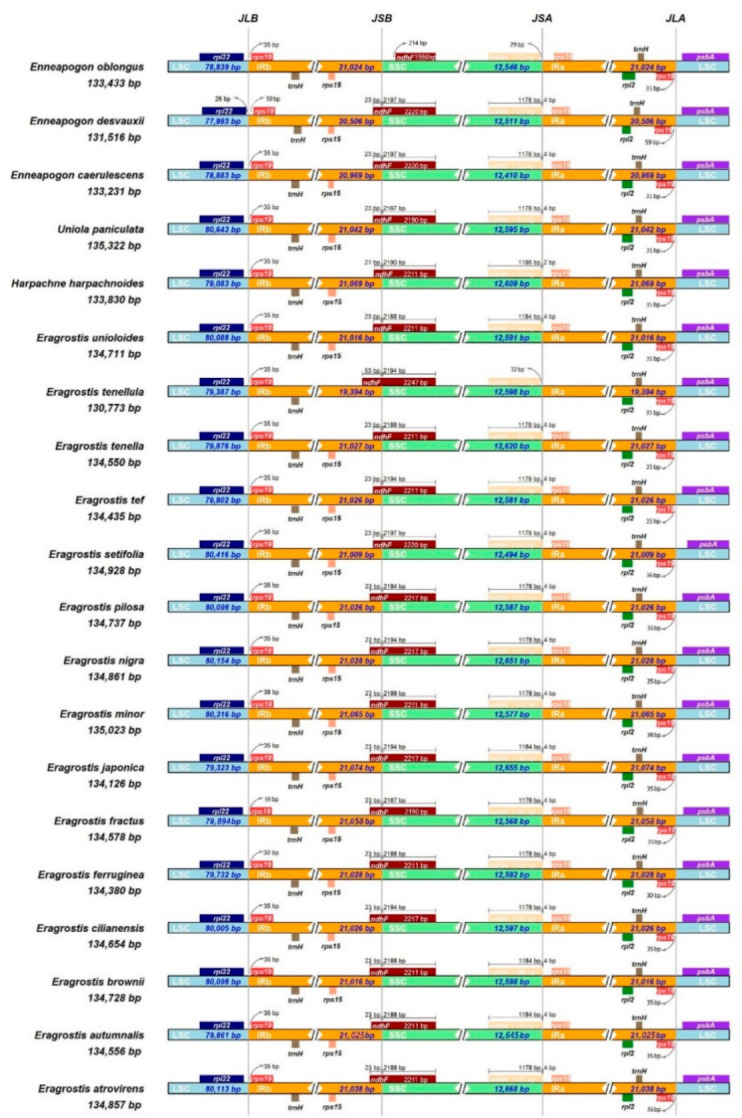
Comparison of the junctions of LSC, SSC, and IR regions among 20 Eragrostideae chloroplast genomes.

The expansion and contraction of the IR region in 20 Eragrostideae chloroplast genomes were analyzed via IRscope. Although the chloroplast genomes of Eragrostideae were highly conserved, there were still some differences in the IR/single cope (SC) border area (Figure 5). The IR_b_/SSC junction (JSB) of all Eragrostideae species (except *E. oblongus*) was located within the *ndhF* gene, and part of this gene was duplicated 21–53 bp in the IR_b_ region. Due to the early termination of the *ndhF* gene in *En. oblongus*, JSB was located in the intergenic region between *rps19* and *rps15*. Similarly, the SSC/IR_a_ junction (JSA) of all Eragrostideae species (except *E. oblongus* and *E. tenellula*) was located within the *ndhH* gene, and this gene extended 2–4 bp into the IR_a_ region. Unlike the above situation, JSA in *E. oblongus* and *E. tenellula* did not extend to the IR_a_ region, so it was located in the intergenic region between *ndhF* and *rps15*. The LSC/IR_b_ junction (JLB) was located between *rpl22* and *rps19*. The fragment size of *rpl22*-*rps19*, located in IR_b_ region was 30 bp in *E. ferruginea*, 36 bp in *E. setifolia*, 38 bp in *E. minor*, 59 bp in *E. desvauxii*, and 35 bp in the remaining species. The LSC/IR_a_ junction (JLA) was located between *rps19* and *psbA*. The fragment size of *rps19*-*psbA* located in IR_a_ region was 30 bp in *E. ferruginea*, 36 bp in *E. setifolia*, 38 bp in *E. minor*, 59 bp in *En. desvauxii*, and 35 bp in remaining species.

### 2.5. Comparative Genome Analysis and Identification of Hypervariable Regions

**Figure 6:**
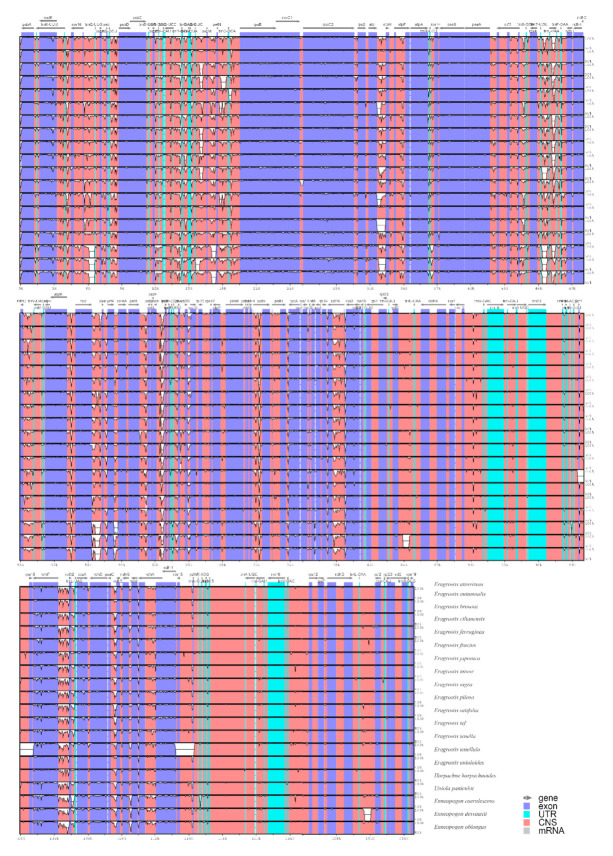
Comparison of 20 Eragrostideae chloroplast genomes using mVISTA alignment program with *E. atrovirens* as a reference. Genome regions are color-coded: blue blocks, exons of protein-coding genes (exon); sky-blue blocks, tRNA and rRNA genes; red blocks, conserved noncoding sequences (CNS). The vertical scale represents a 50–100% of identity.

By comparing the complete chloroplast genomes, we can understand the differences in the chloroplast genome sequences between different species. In this study, the mVISTA program was used to align and compare 20 Eragrostideae chloroplast genomes with *E. atrovirens* as a reference (Figure 6). The results showed that all aligned chloroplast genome sequences have a high similarity. The IR regions were more conservative than the LSC region and the SSC region. The noncoding regions had a higher mutation rate than the protein-coding regions, and the intergenic spacers (IGS) were particularly prominent.

**Figure 7:**
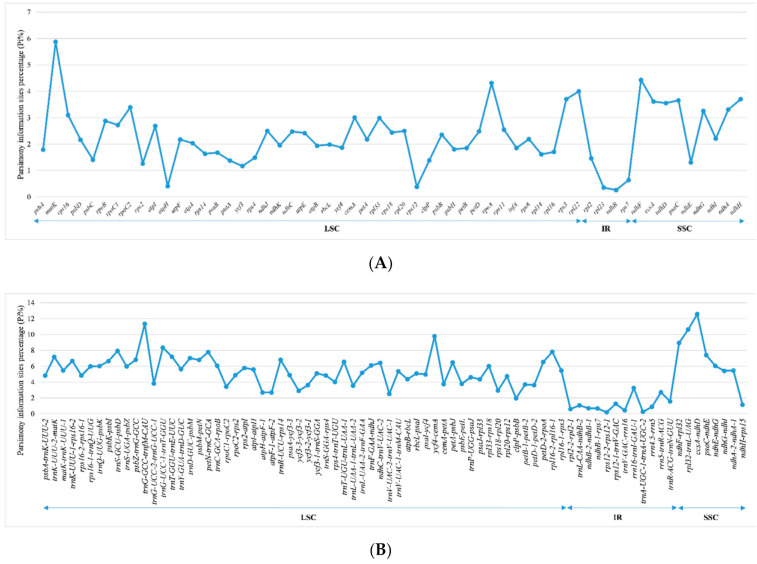
Comparison of percentage of parsimony information sites (Pi%) in 20 Eragrostideae chloroplast genomes. (**A**) Pi% values among coding sequences (CDS); (**B**) Pi% values among noncoding regions (IGS and intron regions).

In order to further identify regions with high mutations, we performed single nucleotide polymorphism (SNP) analysis on the selected CDS and noncoding regions using MEGA v7.0.26, and counted the number of mutation sites and parsimony information sites. Then, the percentage of corresponding parsimony information sites (Pi%) was calculated. We screened 137 regions for analysis, including 58 CDS regions, 64 IGS regions, and 15 intron regions. In the 58 CDS regions, Pi% values ranged from 0.2609 (*ndhB*) to 5.8751 (*matK*). Among them, *rpl22*, *rpoA*, *ndhF*, and *matK* had significantly higher Pi% values (Pi% ≥ 4; Figure 7A). Correspondingly, in 79 noncoding regions, the Pi% values ranged from 0.1572 (*trnI*-*CAU-ycf2*) to 12.5604 (*ccsA-ndhD*). Six of these regions (i.e., *trnG*-*UCC-trnT*-*GGU*, *ndhF-rpl32*, *ycf4-cemA*, *rpl32-trnL*-*UAG*, *trnG*-*GCC-trnfM*-*CAU*, and *ccsA-ndhD*) also showed quite high Pi% values (Pi% > 8; Figure 7B). From the results, the average value of Pi% of the noncoding regions (4.8829) was about twice higher than that of the CDS regions (2.2949), indicating that the noncoding regions were more hypervariable than the CDS regions. Moreover, compared with the SC regions, the Pi% values of the IR regions were lower and relatively more conservative.

### 2.6. Phylogenetic Analysis of Eragrostideae

**Figure 8:**
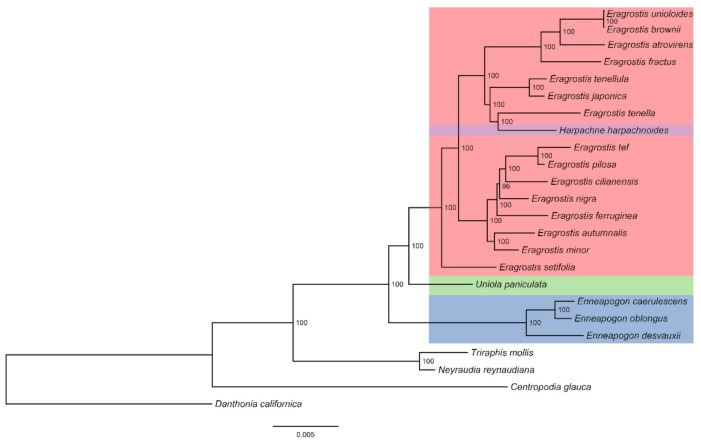
The maximum likelihood phylogenetic tree of 20 Eragrostideae species based on the complete chloroplast genomes. The numbers next to the branches are bootstrap support (BS) values.

In this study, similar topologies were observed in maximum likelihood (ML) trees of seven datasets (complete chloroplast genomes, coding sequences, noncoding sequences, hypervariable regions, LSC regions, SSC regions, and IR regions) among 20 Eragrostideae species (Figure 8 and Figures S2–S7). In general, *Eragrostis*—including *H. harpachnoides*—showed a sister relationship with *Uniola*, and they were sisters to *Enneapogon*. *E. caerulescens*, *E. desvauxii*, and *E. oblongus* formed a monophyletic cluster. In *Eragrostis*, *E. setifolia* diverged first. It showed a monophyletic relationship with the other *Eragrostis* species. *E. mionor* and *E. autumnalis* formed a monophyletic cluster, which was a sister to the other five *Eragrostis* species (*E. tef*, *E. pilosa*, *E. cilianensis*, *E. nigra*, and *E. ferruginea*). The monophyletic cluster comprising *E. japonica* and *E. tenellula* was a sister to the cluster composed of *H. harpachnoides* and *E. tenella*, and both were sisters to the other four *Eragrostis* species (*E. unioloides*, *E. brownie*, *E. atrovirens*, and *E. fractus*). *H. harpachnoides* was embedded in *Eragrostis* with high bootstrap values in all ML analyses (BS > 95%; Figure 8 and Figures S2–S7). In addition, we anatomized the morphology of *H. harpachnoides* and compared it with its sister group *E. tenella* in the phylogenetic tree we generated (Figure 1). In the early taxonomic period, *Harpachne* was recognized as a separate genus due to its racemes being completely different from the panicles of *Eragrostis* (Figure 1A). However, for *E. japonica*, *E. tenellula*, and *E. tenella*, florets disarticulated from above, moved downward, and fell together with the rachilla joints, and an analogous character—that spikelets fall entire together with pedicel—is found in *H. harpachnoides* (Figure 1B). Furthermore, *Harpachne* has characteristics including a ciliated ligule and three-veined lemmas (Figure 1C), which are very similar to other *Eragrostis* species.

## 3. Discussion

### 3.1. Basic Information on the Chloroplast Genomes of Eragrostideae

Chloroplast genomes have the characteristics of small size and a highly conserved structure [4,5]. In this study, chloroplast genomes were conservative in genome size, gene number, and GC content among 20 Eragrostideae species (Table 2), which was consistent with previous Eragrostideae plastome studies [3]. The chloroplast genomes of these species are approximately 134 kb in genome size (Figure S1). The GC content in each species was ca. 38%. Compared with other regions, the IR region had the highest GC content, possibly due to the fact may be because all rRNAs are located in this region [26]. In terms of the gene numbers, protein-coding genes had a small difference (83–87). The number of rRNA and tRNA genes were eight and 38, respectively. Due to the presence of mutations, insertions, and deletions, *rps15* was not annotated in *E. tenellula*. A previous study reported the loss of *accD*, *ycf1*, and *ycf2* genes in the family Poaceae [7]. The gene *accD* might be a useful molecular marker for phylogenetic analysis of land plants and is essential for leaf development [27]. In our study, we found that the *accD* gene had completely degraded in all species. Furthermore, in the case of *ycf1* and *ycf2* loss, there has been a progressive degradation of the gene sequences because different lengths of *ycf1* and *ycf2* were found in our study. The *ycf1* gene has only been annotated in seven species, and retained segments of different lengths range from 78 to 135 bp. The *ycf2* gene has retained segments of different lengths ranging from 105 to 477 bp. Both *ycf1* and *ycf2* have been reported to be essential genes in plants, but their functions are unclear [28,29]. Intron loss of *clpP* and *rpoC1* was detected in all 20 Eragrostideae species. Gene and intron losses can lead to a decrease in chloroplast genome size.

Repeat sequences are not only hotspots where mutations such as nucleotide substitutions and insertion deletions occur, but also very important in phylogenetic research [30]. Repeat sequences are one of the most effective methods to study the origin and evolution of species at the molecular level [31,32]. In this study, 933 repeats were identified in 20 Eragrostideae chloroplast genomes and divided into four types: forward, palindromic, reverse, and complementary. Most of the repeats were forward and reverse repeats. Like most chloroplast genomes of angiosperms [33], most of repeat sequences in the chloroplast genome of Eragrostideae were located in noncoding regions. All the identified repeats in this study may be useful in population genetics studies of these 20 species in the future. SSRs are tandemly repeated DNA sequences, which are widely distributed in the genomes of eukaryotes. The SSRs in the plant chloroplast genomes are rich in genetic variation and have been widely proven as a high-resolution tool for revealing chloroplast genome variation [8]. We detected 943 SSRs in 20 Eragrostideae chloroplast genomes. Similar to the previously reported chloroplast genomes of angiosperms [8,34,35], the predominant type of SSRs were mono-nucleotides, of which, A or T repeats account for the majority. The SSRs detected in the Eragrostideae chloroplast genomes were of great significance for the phylogenetic research and classification of Eragrostideae plants.

Codon usage bias (CUB) is widespread in animals, plants, bacteria, and fungi, reflecting different pressures on different genes or genomes in the evolutionary process. CUB is specific among different species and even between different genes within a species, which is the result of the combined effects of mutation, selection, and drift in the long-term evolution of genes and species [36,37,38]. The results of this study indicated that the CUB of the Eragrostideae chloroplast genomes was weak. Base composition is one of the most pervasive effects of codon usage. In this study, GC content was highly conserved. It is consistent with previously reported Poaceae plastomes that all the 20 plastomes had similar codon usage patterns and preferred to use A/T-terminated codons (Table 4 and Table S1) [39,40]. In all species, nearly all the amino acid codons had a bias (RSCU > 1 or RSCU < 1), except for methionine (AUG, RSCU = 1) and tryptophan (UGG, RSCU = 1). This study can lay the foundation for further research and application of chloroplast genome codons in Eragrostideae.

In previous studies [41,42], the phylogenetic evolution of the chloroplast genome structure in Poaceae plants was reported, and it was found that the LSC/IR boundary had expanded and caused *rps19* and *trnH* to move to the IR region. In addition, the border of SSC/IR_a_ in the ancestors of Poaceae had expanded, resulting in *rps15* being located in the IR region. On the PACMAD (Panicoideae, Aristidoideae, Chloridoideae, Micrairoideae, Arundinoideae, and Danthonioideae) clade of Poaceae, a part of the *ndhF* gene was duplicated at the IR_b_/SSC border, resulting in the border being located inside the *ndhF* gene [43]. In our present study, this phenomenon was observed in most species, with the exception of *En. oblongus* (Figure 5). The differences in the chloroplast genome length (130,773–135,322 bp) of different Eragrostideae plants were mainly caused by the expansion and contraction of the IR region. In general, the contraction and expansion of the IR regions are relatively common evolutionary events in plants and may play an important role in the evolution of plants [44,45].

### 3.2. Phylogenetically Informative Markers

With the continuous deepening study of plant chloroplast genomes, comparative analysis of chloroplast genomes has been paid more and more attention by researchers. Compared with other molecular markers, SNPs have the characteristics of high resolution and genetic stability. In addition, SNPs are unevenly distributed, and most of them are located in noncoding regions [9,46]. In this study, comparative analysis of complete chloroplast genomes in 20 Eragrostideae species showed that IR regions were more conservative than SC regions (Figure 6), and comparisons of the percentage of parsimony information sites confirmed that noncoding regions had higher Pi% than protein-coding genes. A total of 10 regions (*rpl22*, *rpoA*, *ndhF*, *matK*, *trnG*-*UCC-trnT*-*GGU*, *ndhF-rpl32*, *ycf4-cemA*, *rpl32-trnL*-*UAG*, *trnG*-*GCC-trnfM*-*CAU*, and *ccsA*-*ndhD*) with high Pi% were detected, of which, four regions (*rpl22*, *rpoA*, *ndhF*, and *matK*) were located in the coding regions and the rest (*trnG*-*UCC-trnT*-*GGU*, *ndhF-rpl32*, *ycf4-cemA*, *rpl32-trnL*-*UAG*, *trnG-GCC-trnfM-CAU*, and *ccsA-ndhD*) were located in the noncoding regions (Figure 7). Among the 10 potential phylogenetic informative markers, the region *ndhF-rpl32* has been reported as a highly variable marker to study phylogenetic relationships among Eragrostideae species [3]. Understanding and using these variation hotspots is not only helpful for understanding the evolutionary characteristics of the Eragrostideae chloroplast genomes, but also can design molecular markers to provide a data basis for the classification and phylogeny of Eragrostideae.

### 3.3. Phylogenetic Relationships of Eragrostideae

In this study, Eragrostideae were divided into three clades (*Eragrostis*-*Harpachne* clade, *Uniola* clade, and *Enneapogon* clade), representing the three subtribes (Eragrostidinae, Uniolinae, Cotteinae). This was consistent with most previous studies on the phylogeny of Eragrostideae [12,13,14]. *Eragrostis* is the most widely distributed genus with the largest number of species in Eragrostideae, and the interspecies phylogenetic relationships are complicated. Many scholars advocated that several small genera such as *Acamptoclados*, *Cladoraphis*, *Diandrochloa*, *Ectrosia*, *Psammagrostis*, *Harpachne*, and *Neeragrostis* should be reclassified into *Eragrostis* [21,23,47]. In our study, we can intuitively see from all the ML trees that *H. harpachnoides* was sister to *E. tenella* with high support (BS > 95%; Figure 8 and Figures S2–S7). *H. harpachnoides* was embedded within *Eragrostis*, which was consistent with a previous study using the ITS and plastid sequences [12]. Morphologically, the raceme is a simplified structure of the panicle. In addition, *H. harpachnoides* is similar or even identical to the species of *Eragrostis* in the characteristics of spikelet drop patterns, ligule, and lemma. Based on the morphological and molecular evidence from this study, we suggest the reclassification of *Eragrostis*, including *Harpachne*. Few studies have been conducted on the phylogenetic relationship among *Eragrostis* species [3,21,23]. In our research, similar topologies were observed in the phylogenetic analysis of Eragrostideae based on different datasets of complete chloroplast genomes. Our study found that *E. tef* was a sister to *E. polisa*, and *E. minor* was a sister to *E. autumnalis*, which was consistent with a previous study [3]. In addition, our phylogenetic tree supported the clade comprising *H. harpachnoides* and *E. tenella* to be the sister to the clade composed of *E. japonica* and *E. tenellula*. The same result was obtained in previous studies by using nuclear and chloroplast gene data [24,48]. The interspecies relationships of *Eragrostis* were well resolved based on complete chloroplast genomes. This study indicated that complete chloroplast genomes could be used as super-barcodes to resolve the intergeneric and interspecies relationships within Eragrostideae. Moreover, broad sampling and more evidence from morphology and genomes will be necessary for further study of the interspecies relationships within Eragrostideae.

## 4. Materials and Methods

### 4.1. Plant Material, DNA Extraction, and Sequencing

Chloroplast genomes of 11 *Eragrostis* species, *Enneapogon desvauxii*, and *Harpachne harpachnoides* were newly sequenced. Plant material used in this study was deposited at the herbarium of the College of Life Sciences, Shandong Normal University. The voucher specimen information is presented in Table 1. Total genomic DNA was extracted by the modified CTAB method [49]. The total genomic DNA was used for library preparation and paired-end (PE) sequencing by the Illumina Novaseq instrument at Novogene (Beijing, China). Plastomes of other 11 species were downloaded from GenBank (*E. minor* (NC_029412), *E. setifolia* (NC_042832), *E. tef* (NC_029413), *E. tenellula* (NC_042833), *E. caerulescens* (NC_042837), *E. oblongus* (NC_036682), *U. paniculata* (NC_036709), *Neyraudia reynaudiana* (NC_024262), *Triraphis mollis* (NC_042835), *Centropodia glauca* (NC_029411), and *Danthonia californica* (NC_025232)).

### 4.2. Genome Assembly and Annotation

We assembled the chloroplast genome through Organelle Genome Assembler (OGA) [50], as described in Qu et al. [51]. Annotation was performed by using Plastid Genome Annotator (PGA) [52]. Geneious v8.0.2 was used for manual annotation correction [53]. The circular maps for newly sequenced plastomes were generated using the OGDRAW tool [54].

### 4.3. Genome Structure and Expansion and Contraction of IR Region

The chloroplast genome structure, such as gene length, gene number, GC content, intron number were summarized and comparatively analyzed by Geneious v8.0.2. The expansion and contraction of IR regions were analyzed by IRscope [55], coupled with manual modification.

### 4.4. Repeat Sequences and SSR Analysis

The size and position of the repeat sequences were detected using REPuter [56] with the following parameters: hamming distance of 3 and minimum repeat size of 30 bp [56]. MISA script [57] was used to detect SSR, and the minimum number of repeats were set as 10, 6, 5, 5, 5, and 5 for mono-, di-, tri-, tetra-, penta-, and hexanucleotide SSRs, respectively.

### 4.5. Codon Usage

Codons encoding the same amino acid are called synonymous codons, and the difference in use frequency of synonymous codons is the CUB. In order to ensure the accuracy of the results, we eliminated sequences less than 300 bp before codon analysis [58,59]. Then, the codon usage frequency was calculated using codonW v1.4.2 [60]. We also analyzed the effective number of codons (ENC) [61] and relative synonymous codon usage (RSCU). ENC refers to the effective number of codons, and the range of its theoretical value is 20–61, representing the strength of codon bias. The larger the value, the weaker the codon bias. RSCU refers to the relative probability between synonymous codons encoding corresponding amino acids for a particular codon. If there is no preference for the use of codons, the RSCU value of the codon is equal to 1.00. When the RSCU value of a codon is greater than 1.00, it indicates that the frequency of the codon use is relatively high, and vice versa.

### 4.6. Comparative Genome Analysis and Divergent Hotspot Regions

mVISTA [62] is a commonly used comparative chloroplast genome map-drawing program, but the input file needs to meet the format requirements of mVISTA. For comparative analysis, a script [63] was used to convert GenBank annotation files to mVISTA format files. Then, we aligned the complete chloroplast genomes using the mVISTA program with the Shuffle-LAGAN mode, with *E. atrovirens* as a reference [62].

Single nucleotide polymorphism (SNP) mainly refers to a DNA sequence polymorphism caused by single nucleotide variation at the genome level. We counted and calculated the percentage of parsimony information sites (Pi%) of the selected CDS and noncoding regions by using MEGA v7.0.26 [64]. The screening conditions were as follows: (a) sequence length > 200 bp; (b) variable sites and parsimony information sites > 0 [65].

### 4.7. Phylogenetic Analysis

In this study, we downloaded the complete chloroplast genomes of seven Eragrostideae species (Chloridoideae), one Centropodieae species (Chloridoideae), two Triraphideae species (Chloridoideae), and one Danthonieae species (Danthonioideae) from GenBank. *D. californica* was set as outgroup. Sequences were aligned using MAFFT v7.313 [66] with default parameters. The ML trees [1] were reconstructed by RAxML v8.0.26 with the GTRGAMMA substitution model and 1000 bootstrap replicates [67]. Seven trees were reconstructed based on following seven data sets: (1) complete chloroplast genome sequences; (2) coding sequences; (3) noncoding sequences; (4) hypervariable regions (*rpl22*, *rpoA*, *ndhF*, *matK*, *trnG*-*UCC-trnT*-*GGU*, *ndhF-rpl32*, *ycf4-cemA*, *rpl32-trnL*-*UAG*, *trnG*-*GCC-trnfM*-*CAU*, and *ccsA-ndhD*); (5) LSC regions; (6) SSC regions; (7) IR regions.

## 5. Conclusions

In this study, 13 Eragrostideae chloroplast genomes were assembled. Combining the downloaded sequences, a total of 20 Eragrostideae chloroplast genomes were collected. All the plastomes were conserved in structure, gene content, gene order, and IR boundaries. Repeats and SSRs were identified, which are important to study chloroplast genome evolution. By examining parsimony information sites, 10 highly variable regions were identified, which can be used as candidate molecular markers for phylogenetic and population genetics study of Eragrostideae. Our phylogenetic analysis found that three species of *Enneapogon* formed a monophyletic clade. *Enneapogon* diverged first, and *Eragrostis,* including *Harpachne,* is the sister to *Uniola*. In addition, the interspecies relationships of *Eragrostis* were well resolved. *E. setifolia* was suggested to be an early-diverging species. Furthermore, *H. harpachnoides* was considered as a species of *Eragrostis* based on morphological and molecular evidence. This is the first time that the complete chloroplast genomes support the clustering of *Harpachne* into *Eragrostis*. Our results presented here, provide helpful insights into the phylogenetic study of Eragrostideae species based on complete chloroplast genomes. Moreover, broad sampling and more evidence will be necessary for further study into the relationships of Eragrostideae.

## Figures and Tables

**Table 1:** Voucher specimen information of newly sequenced 13 Eragrostideae species.

Species	Collecting Locations	GPS	Voucher Specimen Number	GenBank Accession Number
*Eragrostis atrovirens*	Guangdong, China	113°46″ E, 23°29′ N	SDNU101	MW255512
*E. autumnalis*	Gansu, China	105°53′ E, 34°34′ N	SDNU012	MW255513
*E. brownii*	Guangdong, China	111°57′ E, 22°42′ N	SDNU022	MW255514
*E. cilianensis*	Shandong, China	118°36′ E, 36°12′ N	SDNU235	MW255515
*E. ferruginea*	Shandong, China	117°20′ E, 36°29′ N	SDNU002	MW255517
*E. fractus*	Yunnan, China	100°11′ E, 25°38′ N	SDNU184	MW255518
*E. japonica*	Guangdong, China	111°57′ E, 22°42′ N	SDNU013	MW255519
*E. nigra*	Yunnan, China	100°11′ E, 25°38′ N	SDNU183	MW255521
*E. pilosa*	Guangdong, China	113°46″ E, 23°29′ N	SDNU087	MW255523
*E. tenella*	Guangdong, China	111°57′ E, 22°42′ N	SDNU011	MW255525
*E. unioloides*	Guangdong, China	113°46′ E, 23°29′ N	SDNU003	MW255526
*Harpachne harpachnoides*	Yunnan, China	100°11′ E, 25°38′ N	SDNU088	MW255527
*Enneapogon desvauxii*	Inner Mongolia, China	111°35′ E, 40°51′ N	SDNU046	MW255511

**Table 2:** Chloroplast genome characteristics of 20 Eragrostideae species.

Species	Genome Size	LSC Region	IR Region	SSC Region	GC Content (%)				Number of Genes			
	(bp)	(bp)	(bp)	(bp)	Overall	IR	LSC	SSC	Total	PCGs	rRNAs	tRNAs
*Eragrostis atrovirens*	134,857	80,113	21,038	12,668	38.2	44.0	36.1	32.0	133	87	8	38
*E. autumnalis*	134,556	79,861	21,025	12,645	38.3	44.0	36.2	32.1	131	85	8	38
*E. brownii*	134,728	80,098	21,016	12,598	38.2	44.0	36.1	32.1	131	85	8	38
*E. cilianensis*	134,654	80,005	21,026	12,597	38.2	44.0	36.2	32.1	131	85	8	38
*E. ferruginea*	134,380	79,732	21,028	12,592	38.2	44.0	36.1	32.2	131	85	8	38
*E. fractus*	134,578	79,894	21,058	12,568	38.2	43.9	36.1	32.0	133	87	8	38
*E. japonica*	134,126	79,323	21,074	12,655	38.2	43.9	36.2	32.1	133	87	8	38
*E. minor*	135,023	80,316	21,065	12,577	38.2	44.0	36.2	32.2	131	85	8	38
*E. nigra*	134,861	80,154	21,028	12,651	38.2	44.0	36.2	32.1	131	85	8	38
*E. pilosa*	134,737	80,098	21,026	12,587	38.2	44.0	36.2	32.0	131	85	8	38
*E. setifolia*	134,928	80,416	21,009	12,494	38.3	44.0	36.2	32.4	131	85	8	38
*E. tef*	134,435	79,802	21,026	12,581	38.3	44.0	36.3	32.1	131	85	8	38
*E. tenella*	134,550	79,876	21,027	12,620	38.2	43.9	36.2	32.2	133	87	8	38
*E. tenellula*	130,773	79,387	19,394	12,598	38.4	44.9	36.2	32.2	129	83	8	38
*E. unioloides*	134,711	80,088	21,016	12,591	38.2	44.0	36.1	32.2	131	85	8	38
*Harpachne harpachnoides*	133,830	79,083	21,069	12,609	38.2	43.9	36.2	32.3	133	87	8	38
*Enneapogon* *caerulescens*	133,231	78,883	20,969	12,410	38.3	44.0	36.3	32.3	133	87	8	38
*E. desvauxii*	131,516	77,993	20,506	12,511	38.3	44.1	36.3	32.3	133	87	8	38
*E. oblongus*	133,433	78,839	21,024	12,546	38.3	44.0	36.3	32.3	133	87	8	38
*Uniola paniculata*	135,322	80,643	21,042	12,595	38.3	44.0	36.3	32.4	131	85	8	38

**Table 3:** Numbers of SSRs in 20 Eragrostideae chloroplast genomes.

Species	Total SSRs	Compound SSRs	A/T		C/G		AT/TA		LSC	SSC	IR_a_	IR_b_
			A	T	C	G	AT	TA				
*Eragrostis atrovirens*	40	4	18	21	-	-	-	1	33	3	2	2
*E. autumnalis*	44	5	17	25	1	1	-	-	38	2	2	2
*E. brownii*	52	7	19	28	1	1	1	2	44	6	1	1
*E. cilianensis*	46	4	17	25	1	1	-	2	39	3	2	2
*E. ferruginea*	46	4	20	23	1	1	-	1	37	3	3	3
*E. fractus*	48	7	20	26	-	1	-	1	45	1	1	1
*E. japonica*	51	2	20	27	1	2	-	1	42	3	3	3
*E. minor*	41	5	15	23	1	1	-	1	36	1	2	2
*E. nigra*	53	5	20	29	2	2	-	-	43	4	3	3
*E. pilosa*	50	4	18	26	2	3	-	1	43	1	3	3
*E. setifolia*	40	3	15	23	1	1	-	-	33	2	2	2
*E. tef*	50	7	22	25	1	1	-	1	47	1	1	1
*E. tenella*	56	5	21	30	1	1	1	2	48	2	3	3
*E. tenellula*	49	2	18	27	1	2	-	1	42	3	2	2
*E. unioloides*	52	7	19	29	-	2	-	2	45	5	1	1
*Harpachne harpachnoides*	43	5	15	24	1	1	-	2	36	1	3	3
*Enneapogon caerulescens*	44	2	21	20	-	-	-	3	39	1	2	2
*E. desvauxii*	43	2	17	22	1	1	-	2	37	-	3	3
*E. oblongus*	45	2	21	21	-	-	-	3	40	1	2	2
*Uniola paniculata*	50	8	20	29	-	-	-	1	50	-	-	-

**Table 4:** GC content, codons count, and effective number of codons (ENC) of protein-coding genes in 20 Eragrostideae chloroplast genomes.

Species	GC ^1^	CC ^2^	ENC ^3^
*Eragrostis atrovirens*	0.390	17,169	49.61
*E. autumnalis*	0.389	17,201	49.52
*E. brownii*	0.390	17,171	49.66
*E. cilianensis*	0.389	17,208	49.51
*E. ferruginea*	0.389	17,129	49.56
*E. fractus*	0.389	17,159	49.52
*E. japonica*	0.389	16,999	49.48
*E. minor*	0.390	17,160	49.61
*E. nigra*	0.389	17,208	49.59
*E. pilosa*	0.389	17,210	49.52
*E. setifolia*	0.391	17,149	49.73
*E. tef*	0.390	17,146	49.60
*E. tenella*	0.390	17,169	49.59
*E. tenellula*	0.390	17,123	49.52
*E. unioloides*	0.390	17,171	49.66
*Harpachne harpachnoides*	0.389	17,173	49.55
*Enneapogon* *caerulescens*	0.390	17,158	49.40
*E. desvauxii*	0.390	17,145	49.42
*E. oblongus*	0.390	17,068	49.40
*Uniola* *paniculata*	0.391	17,097	49.80
Average	0.3897	17,151	49.56

^1^ GC content at coding positions; ^2^ Codons count; ^3^ Effective number of codons.

## Data Availability

The data presented in this study are openly available in GeneBank.

## Supplementary Materials

The following are available online at https://www.mdpi.com/2223-7747/10/1/109/s1, Figure S1: Chloroplast genome maps of 13 Eragrostideae species. Genes on the inside of outer circle are transcribed in the clockwise direction and genes on the outside are transcribed in the counterclockwise direction. The dashed darker gray area in the inner circle indicates GC content, while the lighter gray area shows AT content. IR = inverted repeat; SSC = small single copy; LSC = large single copy. Figure S2: The ML tree of 20 Eragrostideae species based on the coding sequences. The numbers next to the branches are bootstrap support values. Figure S3: The ML tree of 20 Eragrostideae species based on the noncoding sequences. The numbers next to the branches are bootstrap support values. Figure S4: The ML tree of 20 Eragrostideae species based on the hypervariable regions (*rpl22*, *rpoA*, *ndhF*, *matK*, *trnG*-*UCC*-*trnT*-*GGU*, *ndhF-rpl32*, *ycf4-cemA*, *rpl32-trnL*-*UAG*, *trnG*-*GCC-trnfM*-*CAU*, and *ccsA-ndhD*). The numbers next to the branches are bootstrap support values. Figure S5: The ML tree of 20 Eragrostideae species based on the LSC regions. The numbers next to the branches are bootstrap support values. Figure S6: The ML tree of 20 Eragrostideae species based on the SSC regions. The numbers next to the branches are bootstrap support values. Figure S7: The ML tree of 20 Eragrostideae species based on the IR regions. The numbers next to the branches are bootstrap support values. Table S1: types of codon encoding amino acids, Table S2: codon usage condition in 20 Eragrostideae chloroplast genomes.
