# Supplementary figures and images for: Comparative and Phylogenetic Analysis of Complete Chloroplast Genomes in Eragrostideae (Chloridoideae, Poaceae)
Source: Plants (Basel). 2021 Jan 6;10(1):109. doi: 10.3390/plants10010109 (PMC7825611; doi:10.3390/plants10010109)

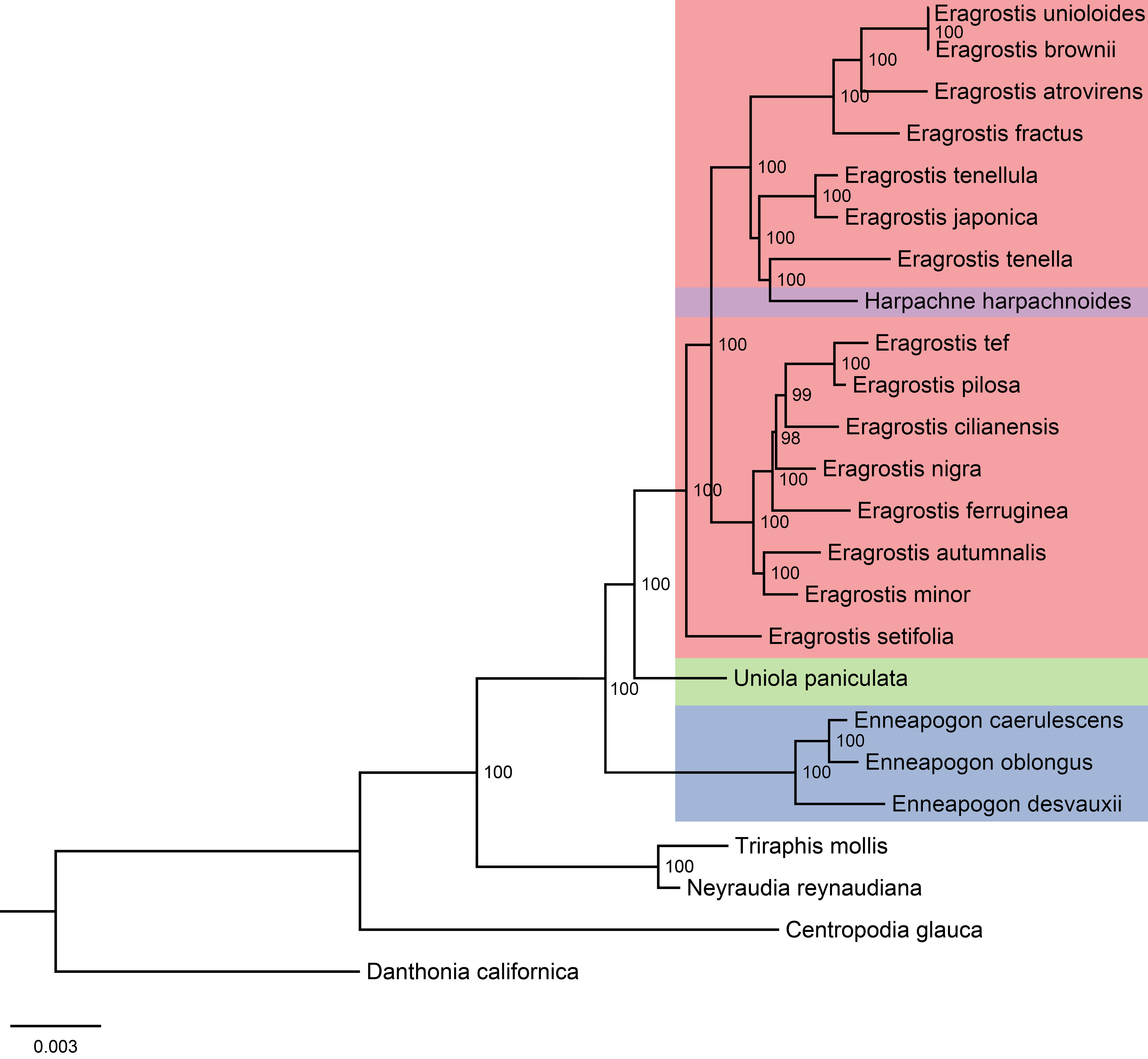

Supplement: Supplementary file 1 [file plants-10-00109-s001.zip › Figure S2.tif]

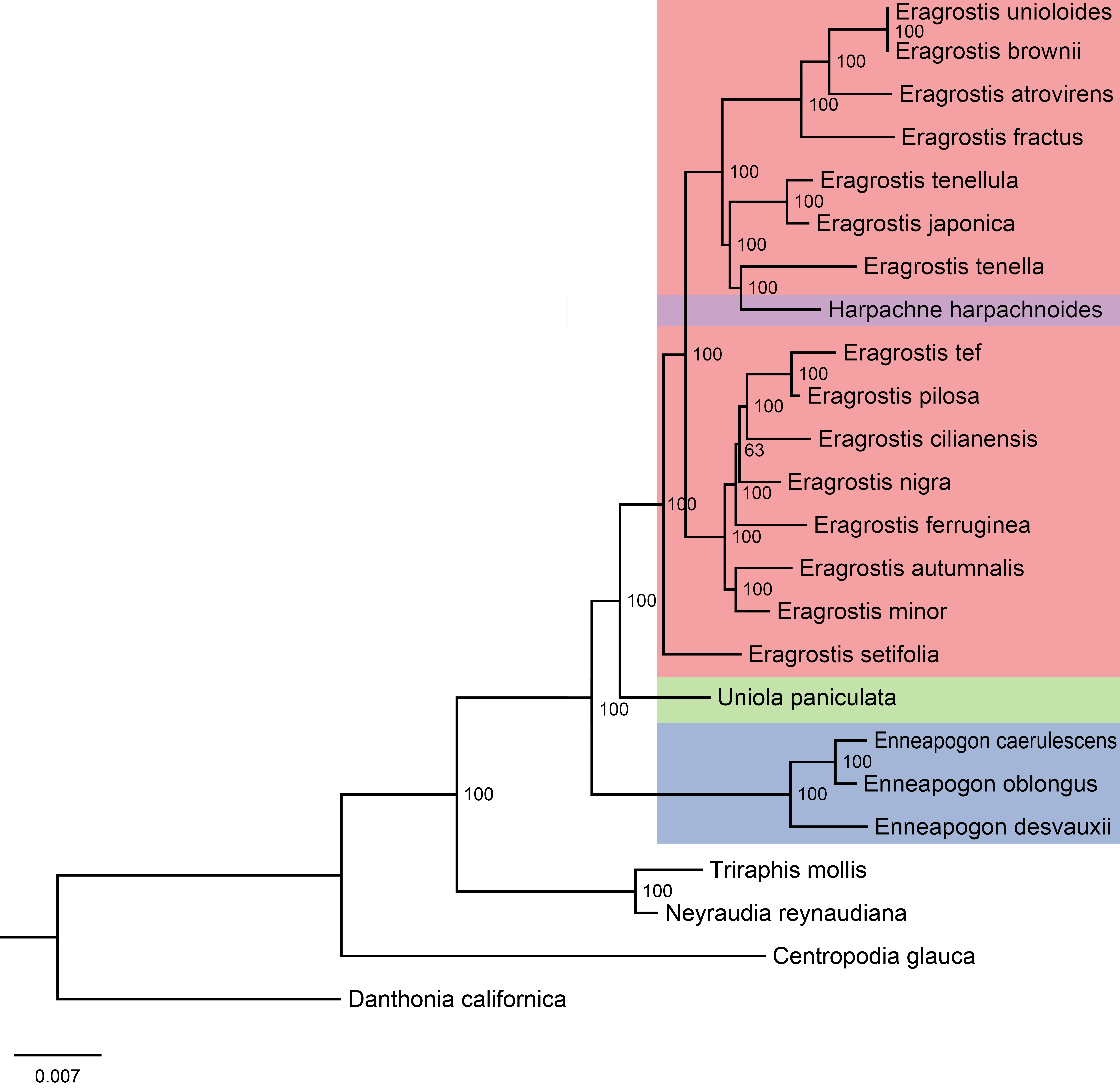

Supplement: Supplementary file 1 [file plants-10-00109-s001.zip › Figure S3.tif]

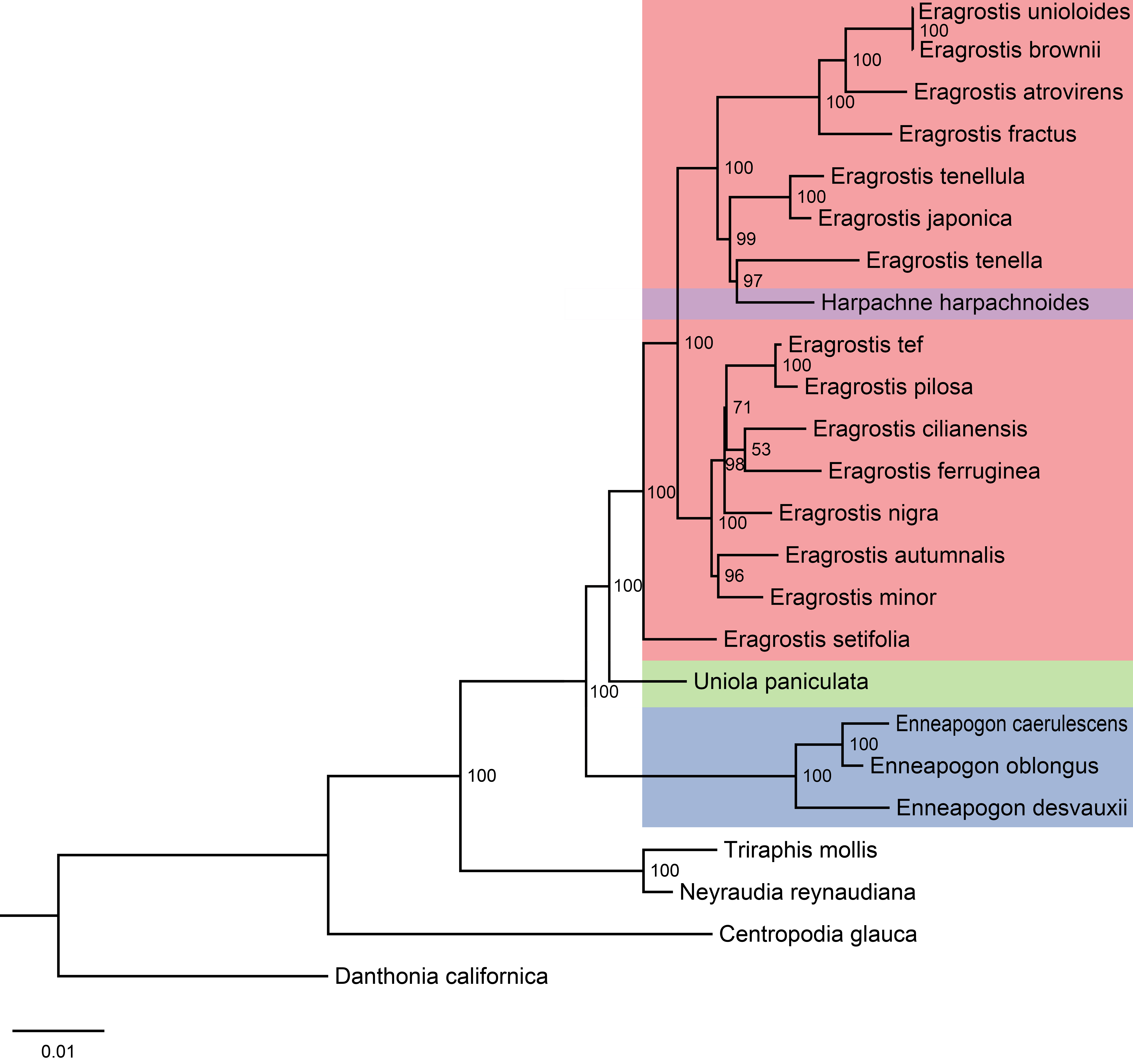

Supplement: Supplementary file 1 [file plants-10-00109-s001.zip › Figure S4.tif]

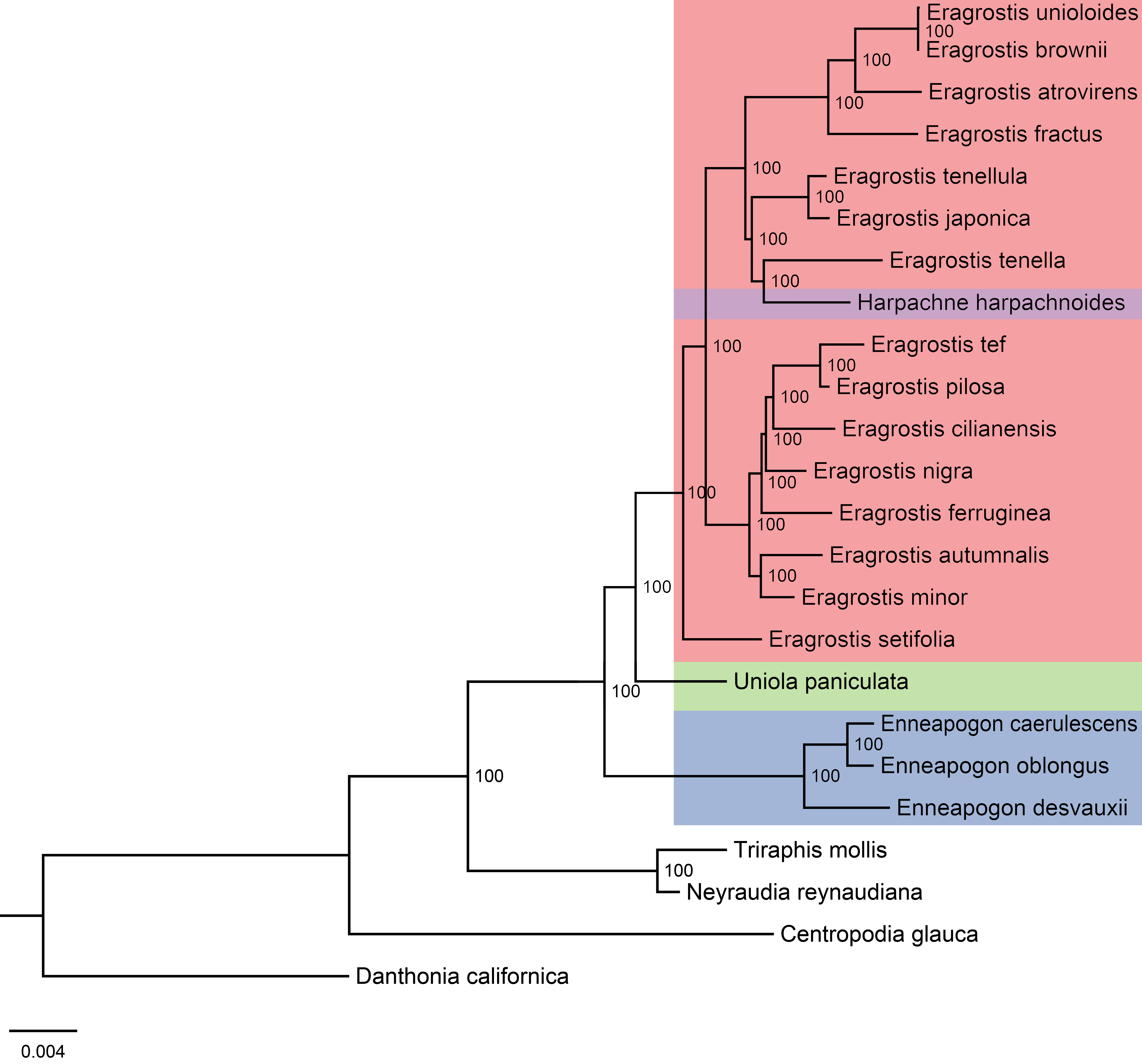

Supplement: Supplementary file 1 [file plants-10-00109-s001.zip › Figure S5.tif]

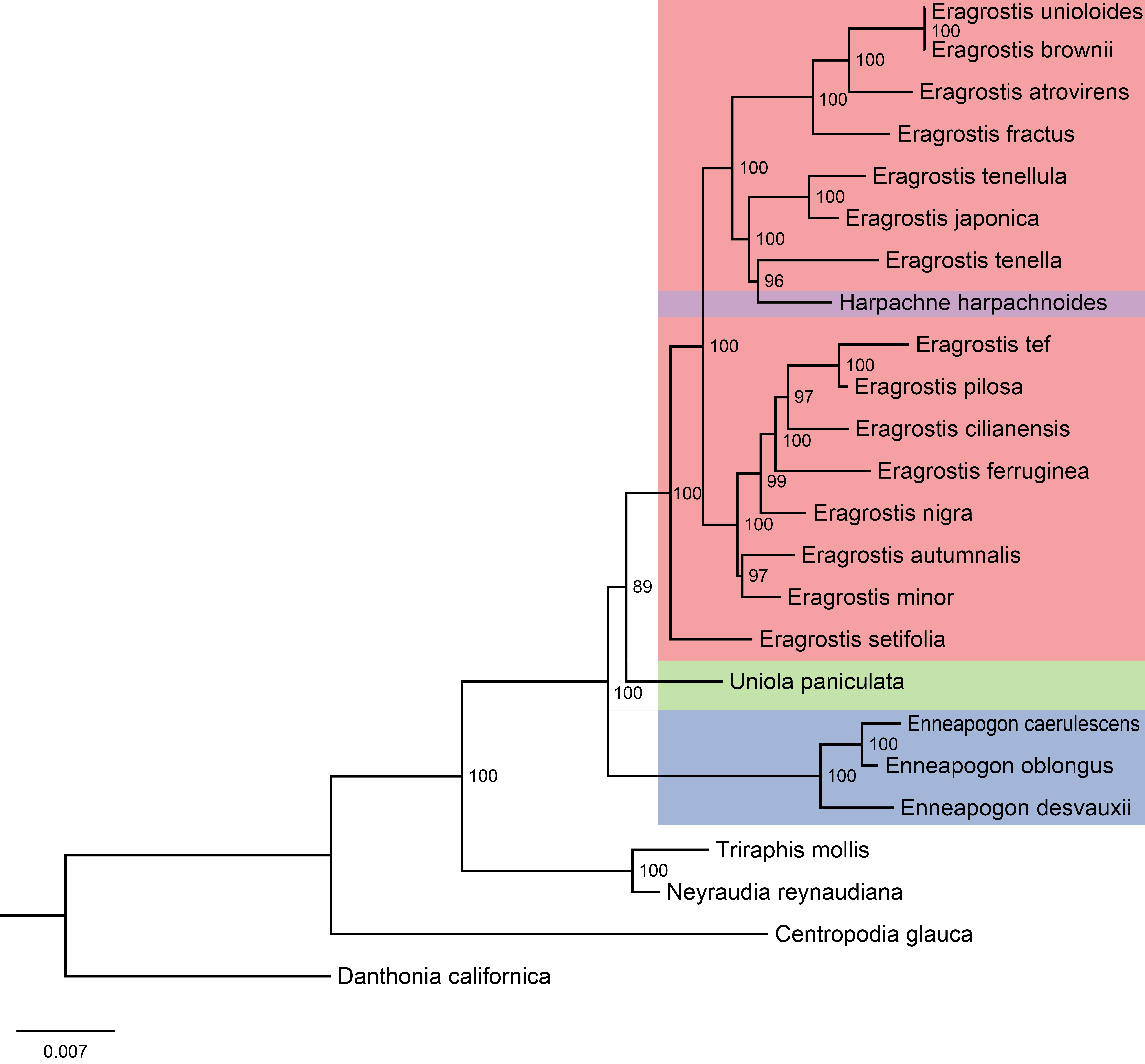

Supplement: Supplementary file 1 [file plants-10-00109-s001.zip › Figure S6.tif]

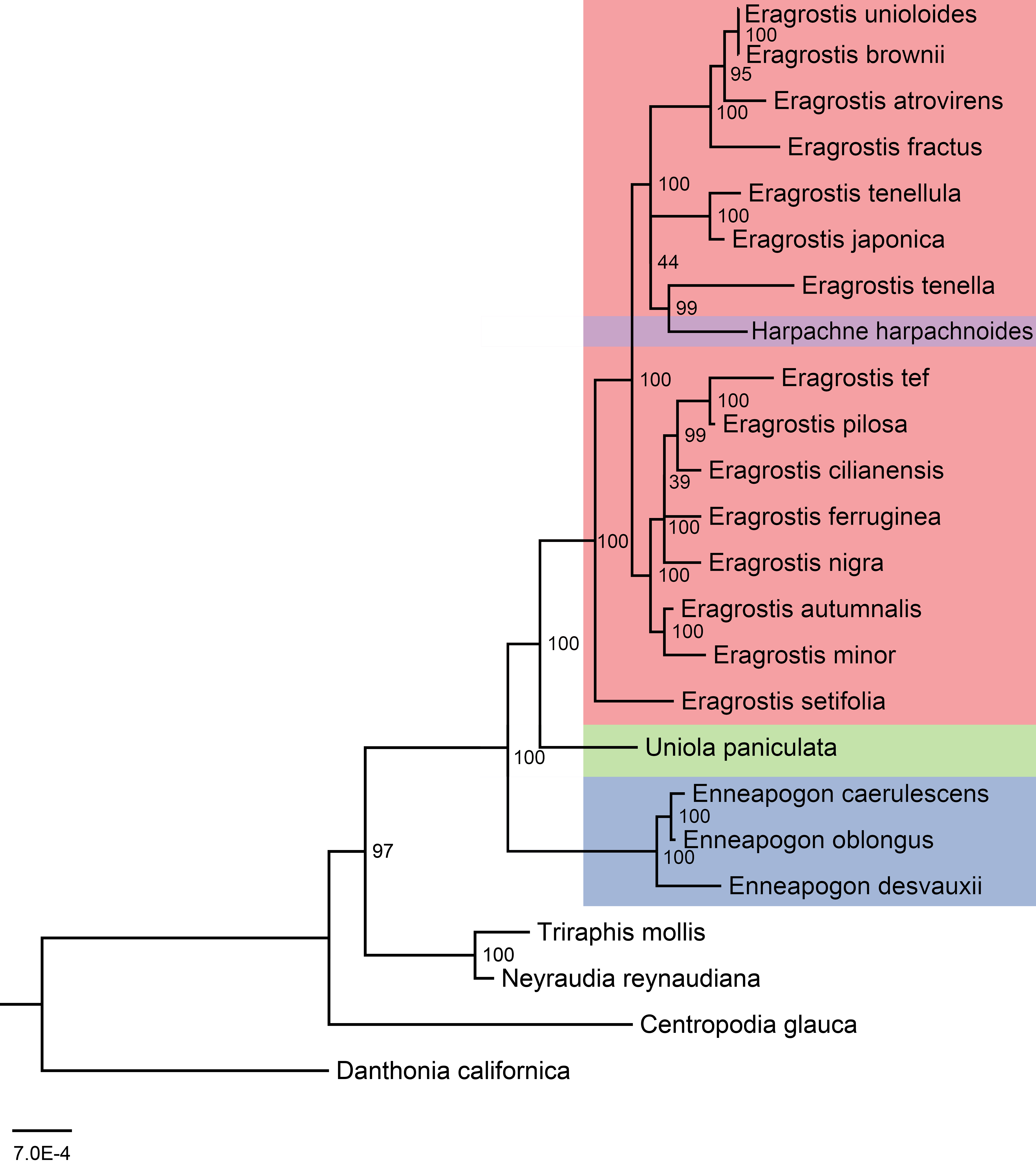

Supplement: Supplementary file 1 [file plants-10-00109-s001.zip › Figure S7.tif]
